# The "Goutte De Lait"

**Published:** 1904-09-24

**Authors:** 


### HOSPITAL ADMINISTRATION.

### CONSTRUCTION AND ECONOMICS.

### THE "GOUTTE DE LAIT."

The arrest of natural increase in the population of France has been the concern of French sociologists, economists, and statesmen for many years. The seriousness of the French condition and tendency can be estimated only by comparing her figures with those relating to other countries in the front rank of the nations. The tables of M. Jacques Berthillon, admittedly authoritative, record the following for the last decade for which statistics are available.

One Year's Average per 1,000 Inhabitants.

| | Births. | Deaths. | Gain. |
|---|---|---|---|
| England ... ... | 30·1 | 18·2 | 13 3 |
| Scotland ... ... | 30·6 | 18·6 | 12·0 |
| Ireland ... ... | 22·8 | 18·1 | 4·9 |
| Germany ... ... | 36·11 | 22·23 | 13·87 |
| Norway ... ... | 30·5 | 16·3 | 14·1 |
| Sweden ... ... | 27 2 | 16·3 | 10·8 |
| Italy ... ... | 34·6 | 24·1 | 10·5 |
| Spain ... ... | 36·9 | 32·4 | 4·5 |
| Austria ... ... | 37·6 | 27·0 | 10·5 |
| Holland ... ... | 32·5 | 18·4 | 14 1 |
| France ... ... | 22·2 | 21·6 | 0·6 |

From these figures several facts stand out in alarming prominence. At the present rate of increase it will take France more than 40 years to add 1,000,000 to her population, while Germany, her natural rival and possible enemy, will, reckoning accumulatively, have increased by 36,000,000 during the same period. Forty years ago the population of France and of Germany were nearly equal. Forty years hence, unless present indications are falsified, Germany will have double the population of France with 10,000,000 to spare. For any country exhibiting such a state of national anæmia, sunk so low in its allegiance to the first individual and national instinct—that of reproduction—there is only one fate, a gradual decline in the scale of importance among the nations of the world. No Frenchman worthy of his heritage can contemplate this prospect without serious apprehension and sincere desire to awaken his countrymen to a sense of the importance of strengthening and increasing the national stock.

A low natality excess may be explained by one or both of two causes—an excessively low birth-rate and an excessively high death-rate. Both factors contribute towards the exceeding poverty of increase in the population of France. An annual birth-rate of only 22·2 per thousand is far below that of any other country. What are its causes?

In the first place, the comfort of the individual and the perpetuation of the race come into implacable conflict. Unions with neither secular nor sacred marriage contracts yield progeny either not at all, or to a degree quite incommensurate to the powers and duties of the parties allied. When a mistress holds the place a wife ought to occupy, children are not desired. The frequency of such unions in France stands as the primal cause of French sterilty; and cause and effect, regarded together, constitute the strongest material argument for the value of marriage as an institution.

Descending further into the general subject one finds that the same result—poverty of increase—arrives by the exigencies of the domestic economy, even when the family is established upon the foundation of a properly sealed marriage contract. The French industrial conditions make it a necessity that the wife should be a bread winner as well as the husband. In Paris, especially, where wages are relatively low, and the cost of living high, the practice of working in factory or shop by the weaker partner, is so universal as to be almost general. This both restricts family increase and raises infant mortality. A consideration of the latter fact brings us into the second main cause of stagnancy in population. When children are born by the working wife their nurture is attended with expense and difficulty. Often they must be sent out to be reared by "eleveuses" or baby farmers, and, although the "Loi Roussel," which makes it compulsory that children so reared should be medically examined at least once a month, restricts the abuses of this system which often come to light in England, it is undesirable in regard to the maintenance of the race. Many working mothers cannot afford the modest expense of an "eleveuse" and a grandmother, the elder children, or a neighbour, must yield the attention which only a mother can properly give. This is the prime factor in the high mortality among French infants.

A French Government commission is supposed to have in hand the consideration of the causes of "depopulation"—so called—and the propounding of remedies for the national cancer. Their work seems to have been spasmodic and the practical results of their deliberations nil. A meeting was held recently, after an interval of fifteen months, since the one immediately preceding. The two official reports which have been issued contain no valuable enlightenment upon the problems supposed to be in process of solution. The enormity of the subject overwhelms the deliberators, and the futility of the search for any remedy carrying the essential of practicability has rendered their work barren.

But quietly, persistently, and effectually, unheralded by the blazon which attends a Parliamentary commission, and leaving the larger issues to be solved as the march of social and industrial progress may direct, a band of men have applied themselves to one department of the problem. While others regard the wilderness of weeds sapping the life of the national organism and spend years in deliberating how and where they shall begin the work of cleaning the soil, these men have turned to one corner of the field and through the agency of the organisations known as "Gouttes de Lait" (*i.e.* drops of milk) are turning furrows which should yield bountiful harvests. The fame of the "Goutte de Lait" movement has spread far beyond the confines of the land where this form of charity first opened its eyes. Medical and public men the world over have welcomed the French pioneers and have watched the work from afar to see if there has come one of those agencies whose potentialities upon the welfare of the human race justify the inauguration of similar enterprises where approximately similar conditions prevail. The experimental stage has passed. The "Gouttes de Lait" have by their results shown the practical value of their constitution and their methods and have established a claim to be regarded as a practical means of conserving human life during the critical months of early infancy.

It was in 1890 that Professor Hergott, of Nancy, founded a charity to encourage poor mothers to suckle their own children instead of having them nursed artificially. The indiscriminate and indiscreet use of the feeding-bottle was, and still is, responsible for a heavy percentage of the high infant mortality of France. At the Nancy charity the children were weighed and examined and the results tabulated. The mothers received a gratuity depending in amount upon the care evidenced by the health and progress of the children and upon the family circumstances. During ten years about £1,000 were distributed among about 2,000 poor mothers. The *per capita* grant was not large but it was sufficient to stimulate mothers to follow the prescribed treatment and the gain in health and the saving of infant life cannot be computed in pounds or francs. The idea expanded and soon another organisation was born. The objective was the same. The methods were slightly different. It was recognised that breast-feeding, while undoubtedly superior to any artificial nurture, was in numerous cases impossible. The daily occupations of the mothers rendered it so. Therefore it was sought to have artifical feeding prosecuted upon as hygienic a basis as its conditions permitted. To M. Budin, of the Charity Hospital in Paris, belongs the honour of having been the first mover in this direction. In 1892, aided by a monetary grant from the "Assistance Publique," he inaugurated an out-patient department for nurslings, and mothers received, under proper medical advice, supplies of sterilised milk for their infants. A few months later there followed Dr. G. Variot at his dispensary in Belleville, one of the poorest quarters of the French capital. The latter has always been essentially a charity dependent upon the gift-offerings of its well-wishers. Then Dr. Dufour of Fècamp entered the same field and christened his organisation the "Goutte de Lait," an expressive designation which speedily took the fancy, was soon applied to all similar charitable institutions and has been adopted into English without translation.

As one watches the mothers bring their babies up to the desks of the doctors in the Belleville Dispensary, certain words recorded of the rebuilding of the walls of Jerusalem spontaneously invade the mind. Of many of the old Jewish builders it is written that they built over against their own doors. Simple words, of which the significance is apt to be missed. Yet how eloquent of the ready acceptance of the first public task that came to hand. Thus with the "Goutte de Lait" doctors. Carrying on his shoulders the responsibilities of a large consulting practice and of the editorship of a French medical journal, Dr. Variot yet manages to give about half of his life to the "Goutte de Lait" crusade. His platform work has been important. He has carried on the propaganda by lectures, drawing poor mothers to the halls by the enticements of musical entertainments and instilling into them the rules which should govern the rearing of their children. His only fee has been the gratification that follows success. Perhaps the tribute of art to the value of the efforts spent in the cause of humanity may be reckoned reward. It was at least recognition. The painter Geoffrey exhibited a fine tableau of the Belleville "Goutte de Lait," with Dr. Variot as the central figure, in the Salon of 1903. The picture was bought by the City of Paris and may be seen in the permanent collection exhibited in the Petit Palais in the Champs Elysée.

*(To be continued.)*

